# Supplementary material for: The social cost of investor distraction: Evidence from institutional cross-blockholding
Source: PLoS One. 2023 Dec 7;18(12):e0286336. doi: 10.1371/journal.pone.0286336 (PMC10703221; doi:10.1371/journal.pone.0286336)
Supplement: S1 Appendix — (DOCX) [file pone.0286336.s001.docx]

**The Social Cost of Investor Distraction: Evidence from Institutional Cross-Blockholding**

**Appendix**

**Table A1**

**Variable Definitions and Data Sources**

| Variable | Definition | Source |
| --- | --- | --- |
| ***Measures of Corporate Social Responsibility*** | | |
| CSR Performance *(CSR)* | CSR performance, where CSR performance equals CSR strength score minus CSR concerns score. | KLD |
| CSR Strength *(STR)* | CSR strength score, which is the sum of community activities, diversity, employee relations, environmental record, and product quality and safety strengths. | KLD |
| CSR Concern *(CON)* | CSR concern score, which is the sum of community activities, diversity, employee relations, environmental record, and product quality and safety concerns. | KLD |
| Community *(COM)* | CSR performance in Community, which equals community strengths minus community concerns. | KLD |
| Workforce diversity *(DIV)* | CSR performance in Diversity, which equals workforce diversity strengths minus workforce diversity concerns. | KLD |
| Employee relations *(EMP)* | CSR performance in Employee relations, which equals employee relations strengths minus employee relations concerns. | KLD |
| Environment impact *(ENV)* | CSR performance in Environment, which equals environment impact strengths minus environment impact concerns. | KLD |
| Product quality *(PRO)* | CSR performance in Product, which equals product quality strengths minus product quality concerns. | KLD |
| Community concerns | Investment controversies, community impact, tax disputes, and other concerns. | KLD |
| Community strengths | Charitable giving, innovative giving, non-US charitable giving, support for housing, support for education, and other strengths. | KLD |
| Workforce diversity concerns | Controversies, non-representation, board diversity, and other concerns. | KLD |
| Workforce diversity strengths | CEO, promotion, board of directors, work/life benefits, women & minority contracting, employment of the disabled, gay & lesbian policies, and other strengths. | KLD |
| Employee relations concerns | Union relations, health and safety concerns, workforce reductions, retirement benefits concerns, and other concerns. | KLD |
| Employee relations strengths | Union relations, cash profit sharing, employee involvement, retirement benefits strengths, health and safety strengths, and other strengths. | KLD |
| Environment impact concerns | Hazardous waste, regulatory problems, ozone depleting chemicals, substantial emissions, agricultural chemicals, climate change, and other concerns. | KLD |
| Environment impact strengths | Beneficial products and services, pollution prevention, recycling, clean energy, and other strengths. | KLD |
| Product quality concerns | Product safety, marketing/contracting, antitrust, and other concerns. | KLD |
| Product quality strengths | Quality, R&D/Innovation, benefits to economically disadvantaged, and other strengths. | KLD |
| *SOCIALS* | The social score in the Sustainalytics Database. | Sustainalytics |
| *AvgSOCIALS* | The 2-year average of social score in the Sustainalytics Database. | Sustainalytics |
| ***Measures of Cross-blockholding*** | | |
| *CROSS_DUM* | An indicator that equals one if the firm is cross-held by institutional investors in any quarter of a year. | Thomson Reuters Institutional (13F) Holdings |
| *AVGNUM* | The average number of peers in the same industry that are held by the same blockholders. | Thomson Reuters Institutional (13F) Holdings |
| *CROSS_OWN* | The total percentage of shares held by cross-blockholders. | Thomson Reuters Institutional (13F) Holdings |
| *NUMCROSS* | The number of unique cross-blockholders. | Thomson Reuters Institutional (13F) Holdings |
| *NUMCONNECT* | The number of peer firms in the same industry that share any common blockholder with the firm. | Thomson Reuters Institutional (13F) Holdings |
| ***Other Variables*** | | |
| *SIZE* | The logarithm of 1 plus the book value of total assets (*#AT*). | COMPUSTAT |
| *TOBINQ* | The ratio of market value to book value (*#AT*), where market value is defined as total assets (*#AT*) minus common equity (*#CEQ*) and deferred taxes (*#TXDB*) plus the market equity *(#PRCC_F* *× #CSHO*). | COMPUSTAT |
| *BLEV* | Long-term debt (*#DLTT*) and debt in current liabilities (*#DLC*) scaled by the book value of total assets (*#AT*). | COMPUSTAT |
| *CAPX* | Capital Expenditures (*#CAPX*) scaled by the book value of total assets (*#AT*). | COMPUSTAT |
| *PPENT* | Property, plant and equipment (*#PPENT*) scaled by the book value of total assets (*#AT*). | COMPUSTAT |
| *EBITDA* | The sum of income before extraordinary (*#IB*), interest and related expense (*#XINT*), income taxes (*#TXT*), depreciation and amortization (*#DP*) scaled by the book value of total assets (*#AT*). | COMPUSTAT |
| *RETA* | Retained earnings (*#RE*) scaled by the book value of total assets (*#AT*). | COMPSTAT |
| *NAN* | The logarithm of 1 plus the arithmetic mean of the 12 monthly numbers of analysts following a firm in a fiscal year. | I\B\E\S |
| *INSTO* | Institutional ownership measured by the percent of shares held by institutional investors. (*INSTOWN_PERC* in WRDS Thomson Reuters Institutional (13f) Holdings Stock Ownership Summary File). | Thomson Reuters Institutional (13F) Holdings |
| *LN_NUM_INST* | The logarithm of 1 plus the arithmetic mean of the 4 quarterly numbers of 13F institutional investors of each firm-year. | Thomson Reuters Institutional (13F) Holdings |
| *Ln(1+CEO Delta)* | The logarithm of 1 plus the CEO’s delta measured as Coles and Naveen (2006). | Excucomp |
| *Ln(1+CEO Total Pay)* | The logarithm of 1 plus the CEO’s total pay (#*TDC1*). | Excucomp |
| *Product Market Fluidity* | Product market fluidity measured by Hoberg, Phillips, and Prabhala’s (2014). | Hoberg-Phillips |
| *Hirfindahl-Hirschman Index* | Herfindahl-Hirschman index based on SIC 2 industry classification. | COMPSTAT |
| *Takeover Risk* | Takeover risk measured as Cremers, Nair, and John (2009). | SDC/COMPUSTAT |
| *Total ESV* | EDGAR search volume (*ESV*) measured by Loughran and McDonald (2017). | James Ryans’ EDGAR Server Log File |
| *Non-IRS ESV* | EDGAR search volume excluding IRS attention. IRS search data come from Bozanic et al. (2019). | James Ryans’ EDGAR Server Log File |
| *ESV Financial* | EDGAR search volume (*ESV*) of firms’ 10-K and 10-Q filings measured by Loughran and McDonald (2017). | James Ryans’ EDGAR Server Log File |
| *ESV Non-Financial* | EDGAR search volume (*ESV*) of firms’ other filings measured by Loughran and McDonald (2017). | James Ryans’ EDGAR Server Log File |
| *%SRI* | The percent of proposals on socially responsible investment (SRI). | ISS Shareholder Proposals |
| *%SRI_PASS* | The percent of passed proposals on socially responsible investment (SRI). | ISS Shareholder Proposals |
| *NUM_SRI* | The number of proposals on socially responsible investment (SRI). | ISS Shareholder Proposals |
| *NUM_SRI_PASS* | The number of passed proposals on socially responsible investment (SRI). | ISS Shareholder Proposals |
|  | | |

**Table A2**

**Institutional Cross-blockholding over the Sample Period**

This table presents the annual average of institutional cross-blockholding measures in each year over the sample period during 1995-2014. Column (1) shows the number of non cross-held firms in the sample. Column (2) shows the number of cross-held firms by year. Column (3) reports the percentage of cross-held firms, which equals the annual average of *CROSS_DUM* by. Columns (4)-(7) report the annual average of *CROSS_OWN*, *AVGNUM*, *NUMCONECT*, *NUMCROSS*, respectively.

|  |  | (1) | (2) | (3) | (4) | (5) | (6) | (7) |
| --- | --- | --- | --- | --- | --- | --- | --- | --- |
| Year | N | *Non cross-held* | *Cross-held* | *Percentage* | *CROSS_OWN* | *AVGNUM* | *NUMCONNECT* | *NUMCROSS* |
|  |  |  |  |  |  |  |  |  |
| 1995 | 164 | 78 | 86 | 52.44% | 0.06 | 1.33 | 1.31 | 0.75 |
| 1996 | 172 | 73 | 99 | 57.56% | 0.06 | 1.17 | 1.37 | 0.84 |
| 1997 | 180 | 77 | 103 | 57.22% | 0.06 | 1.20 | 1.34 | 0.81 |
| 1998 | 151 | 68 | 83 | 54.97% | 0.06 | 1.08 | 1.34 | 0.80 |
| 1999 | 156 | 63 | 93 | 59.62% | 0.06 | 1.56 | 1.47 | 0.93 |
| 2000 | 176 | 66 | 110 | 62.50% | 0.08 | 1.78 | 1.36 | 1.14 |
| 2001 | 339 | 109 | 230 | 67.85% | 0.09 | 2.03 | 1.62 | 1.23 |
| 2002 | 377 | 121 | 256 | 67.90% | 0.09 | 2.08 | 1.59 | 1.23 |
| 2003 | 821 | 276 | 545 | 66.38% | 0.09 | 2.08 | 1.62 | 1.30 |
| 2004 | 894 | 265 | 629 | 70.36% | 0.10 | 2.20 | 1.76 | 1.41 |
| 2005 | 901 | 266 | 635 | 70.48% | 0.10 | 2.20 | 1.71 | 1.44 |
| 2006 | 940 | 275 | 665 | 70.74% | 0.10 | 2.27 | 1.79 | 1.58 |
| 2007 | 1,025 | 266 | 759 | 74.05% | 0.12 | 2.46 | 1.68 | 1.84 |
| 2008 | 1,083 | 282 | 801 | 73.96% | 0.11 | 2.53 | 1.89 | 1.76 |
| 2009 | 1,049 | 252 | 797 | 75.98% | 0.11 | 3.27 | 1.98 | 1.99 |
| 2010 | 1,019 | 312 | 707 | 69.38% | 0.10 | 2.48 | 1.69 | 1.60 |
| 2011 | 889 | 194 | 695 | 78.18% | 0.12 | 4.08 | 2.43 | 1.89 |
| 2012 | 930 | 191 | 739 | 79.46% | 0.13 | 4.36 | 2.56 | 2.07 |
| 2013 | 947 | 188 | 759 | 80.15% | 0.14 | 4.99 | 2.66 | 2.17 |
| 2014 | 899 | 166 | 733 | 81.54% | 0.15 | 5.47 | 2.72 | 2.30 |
|  |  |  |  |  |  |  |  |  |

**Table A3**

**Balance Test of PSM-DID**

This table reports the results of the balance test of the propensity score matching for the robustness tests shown in Panel B of Table 5. The matching variables include firm size (*SIZE*), Tobin’s Q (*TOBINQ*), book leverage (*BLEV*), profitability (*EBITDA*), collateral (*PPENT*), investment (*CAPX*), institutional ownership (*INSTO*), analyst coverage (*NAN*), retained earnings (*RETA*), and the log number of 13F institution investors (*LN_NUM_INST*), industry dummies, and year dummies. Firm characteristics in the balance test include firm size (*SIZE*), Tobin’s Q (*TOBINQ*), book leverage (*BLEV*), profitability (*EBITDA*), collateral (*PPENT*), investment (*CAPX*), institutional ownership (*INSTO*), analyst coverage (*NAN*), retained earnings (*RETA*), and the log number of 13F institution investors (*LN_NUM_INST*). The means of treatment group and control group are shown in Columns (2) and (3), respectively. The differences between the two groups and its corresponding p-value of the equality test are provided in Columns (4) and (5), respectively.

|  | (1) | (2) | (3) | (4) | (5) |
| --- | --- | --- | --- | --- | --- |
| Variables | Un-/Matched | Treated | Control | Diff. | p-value |
|  |  |  |  |  |  |
| *SIZE* | Unmatched | 6.8862 | 6.7623 | 1.9300 | 0.0540* |
|  | Matched | 6.8862 | 6.7797 | 1.2000 | 0.2290 |
|  |  |  |  |  |  |
| *TOBINQ* | Unmatched | 1.8182 | 1.9053 | -1.6400 | 0.1010 |
|  | Matched | 1.8182 | 1.8597 | -0.6400 | 0.5200 |
|  |  |  |  |  |  |
| *BLEV* | Unmatched | 0.1993 | 0.1898 | 1.1000 | 0.2700 |
|  | Matched | 0.1993 | 0.1992 | 0.0000 | 0.9960 |
|  |  |  |  |  |  |
| *EBITDA* | Unmatched | 0.1207 | 0.1190 | 0.2900 | 0.7730 |
|  | Matched | 0.1207 | 0.1187 | 0.2600 | 0.7960 |
|  |  |  |  |  |  |
| *PPENT* | Unmatched | 0.2773 | 0.2384 | 3.8200 | 0.0000*** |
|  | Matched | 0.2773 | 0.2809 | -0.2400 | 0.8140 |
|  |  |  |  |  |  |
| *CAPX* | Unmatched | 0.0569 | 0.0480 | 3.3900 | 0.0010*** |
|  | Matched | 0.0569 | 0.0618 | -1.1200 | 0.2630 |
|  |  |  |  |  |  |
| *INSTO* | Unmatched | 0.8346 | 0.8206 | 1.9000 | 0.0580* |
|  | Matched | 0.8346 | 0.8315 | 0.3400 | 0.7370 |
|  |  |  |  |  |  |
| *NAN* | Unmatched | 2.0723 | 1.9745 | 2.8100 | 0.0050*** |
|  | Matched | 2.0723 | 2.0648 | 0.1600 | 0.8710 |
|  |  |  |  |  |  |
| *RETA* | Unmatched | 0.1310 | 0.1546 | -1.1100 | 0.2670 |
|  | Matched | 0.1310 | 0.1094 | 0.7800 | 0.4380 |
|  |  |  |  |  |  |
| *LN_NUM_INST* | Unmatched | 5.0437 | 4.9721 | 2.6600 | 0.0080*** |
|  | Matched | 5.0437 | 5.0039 | 1.0900 | 0.2760 |

**Table A4**

**Distraction Channel: Evidence from Multivariate OLS Analysis**

This table shows the distraction channel of the relation between CSR and institutional cross-blockholding in multivariate OLS analysis. Panel A shows the relation between institutional cross-blockholding and investor attention. Investor attention is measured by the EDGAR search volume (ESV) measured by Loughran and McDonald (2017). *Non-IRS ESV* denotes the total search volume of non-robot page viewers excluding IRS search records, where IRS search data come from Bozanic et al. (2019). The independent variable, *CROSS_DUM*, is an indicator that equals one if the firm is cross-held in any quarter of the year. Panel B reports the impact of cross-blockholding on CSR across the level of investor attention. The dependent variable is *CSR_t+1_.* A firm is assigned to the High (Low) group if the value of the variable is above (below) the median of the sample. A Wald test is implemented to test the differences of estimated coefficients between the High and the Low group. Model settings and control variables are the same as those in Table 2. Standard errors are clustered by firm. *, **, and *** indicate significance at the 10%, 5%, and 1% levels, respectively. Standard errors are shown in the parentheses.

**Panel A: Cross-blockholding and investor attention**

|  | (1) | (2) | (3) | (4) |
| --- | --- | --- | --- | --- |
| Variables | *Total ESV _t+1_* | *ESV Financial* | *ESV Non-financial* | *Non-IRS ESV* |
|  |  |  |  |  |
| *CROSS_DUM* | -0.0303** | -0.0132 | -0.0232* | -0.0304** |
|  | (0.0125) | (0.0173) | (0.0137) | (0.0125) |
| Constant | 5.8783*** | 4.1836*** | 5.6752*** | 5.8742*** |
|  | (0.1463) | (0.1907) | (0.1754) | (0.1466) |
|  |  |  |  |  |
| Controls | Yes | Yes | Yes | Yes |
| Firm FE | Yes | Yes | Yes | Yes |
| Industry×Year FE | Yes | Yes | Yes | Yes |
| Observations | 11,177 | 11,177 | 11,177 | 11,177 |
| R-squared | 0.9448 | 0.9220 | 0.9015 | 0.9447 |

**Panel B: Effects across investor attention**

|  | (1) | (2) | (3) | (4) |
| --- | --- | --- | --- | --- |
|  | *CSR_t+1_* | *CSR_t+1_* | *CSR_t+1_* | *CSR_t+1_* |
|  |  |  |  |  |
| *CROSS_DUM×High Total ESV* | -0.1073* |  |  |  |
|  | (0.0619) |  |  |  |
| *CROSS_DUM×Low Total ESV* | -0.1947*** |  |  |  |
|  | (0.0515) |  |  |  |
| *CROSS_DUM×High ESV Financial* |  | -0.1042* |  |  |
|  |  | (0.0615) |  |  |
| *CROSS_DUM×Low ESV Financial* |  | -0.1980*** |  |  |
|  |  | (0.0517) |  |  |
| *CROSS_DUM×High ESV Non-financial* |  |  | -0.1140* |  |
|  |  |  | (0.0608) |  |
| *CROSS_DUM×Low ESV Non-financial* |  |  | -0.1898*** |  |
|  |  |  | (0.0512) |  |
| *CROSS_DUM×High Non-IRS ESV* |  |  |  | -0.1078* |
|  |  |  |  | (0.0618) |
| *CROSS_DUM×Low Non-IRS ESV* |  |  |  | -0.1944*** |
|  |  |  |  | (0.0515) |
| Firm FE | Yes | Yes | Yes | Yes |
| Industry×Year FE | Yes | Yes | Yes | Yes |
| Observations | 11,180 | 11,180 | 11,180 | 11,180 |
| R-squared | 0.7072 | 0.7072 | 0.7072 | 0.7072 |
| Difference: High-:Low | 0.0873* | 0.0937* | 0.0757 | 0.0865* |
| p-value of Wald test | [0.0924] | [0.0699] | [0.1206] | [0.0948] |

**Table A5**

**Institutional Cross-blockholding and CSR Performance: Types of Blockholders**

This table presents the cross-sectional effects of institutional cross-blockholding on firms’ CSR performance by types of blockholders. The investor types are classified according to Bushee and Noe (2000) and Bushee (2001). Only observations that can link to Bushee’s institutional investor classification data are included in the tests. The dependent variable is *CSR_t+1_*. The independent variable *CROSS_DUM* is calculated in different types of institutional investors (dedicated investor, quasi-indexer, and transient investor). The control variables are the same as those in Table 2. Firm and industry-year fixed effects are included in the regressions. Industries are classified by Fama-French 48 industries. Standard errors are adjusted for heteroskedasticity and clustered by firm. *, **, and *** indicate significance at the 10%, 5%, and 1% levels, respectively. Standard errors are shown in the parentheses.

|  | Dependent variable= *CSR_t+1_* | | |
| --- | --- | --- | --- |
|  | (1) | (2) | (3) |
|  | Dedicated Investor | Quasi-indexer | Transient Investor |
|  |  |  |  |
| *DED _CROSS_DUM* | 0.0094 |  |  |
|  | (0.1116) |  |  |
| *QXI _CROSS_DUM* |  | -0.0748* |  |
|  |  | (0.0437) |  |
| *TRA _CROSS_DUM* |  |  | -0.2302*** |
|  |  |  | (0.0575) |
| Constant | -0.8386 | -0.8212 | -0.7697 |
|  | (0.6049) | (0.6036) | (0.6029) |
|  |  |  |  |
| Controls | Yes | Yes | Yes |
| Firm FE | Yes | Yes | Yes |
| Industry×Year FE | Yes | Yes | Yes |
| Observations | 8,349 | 8,349 | 8,349 |
| R-squared | 0.7599 | 0.7601 | 0.7608 |
