# Supplementary material for: The social cost of investor distraction: Evidence from institutional cross-blockholding
Source: PLoS One. 2023 Dec 7;18(12):e0286336. doi: 10.1371/journal.pone.0286336 (PMC10703221; doi:10.1371/journal.pone.0286336)
Supplement: Attachment — Submitted filename: Response to Reviewers.docx [file pone.0286336.s002.docx]

Dear Academic Editor, Reviewer #1, and Reviewer #2

Thank you for your feedback and granting us an opportunity to revise the manuscript. We would like to believe that we have addressed all your concerns. We reproduce your comments in italics and write our responses in regular font.

**Authors’ Responses to Academic Editor’s Comments**

*Thank you for submitting your manuscript to PLOS ONE. After careful consideration, we feel that it has merit but does not fully meet PLOS ONE’s publication criteria as it currently stands. Therefore, we invite you to submit a revised version of the manuscript that addresses the points raised during the review process.*

*After careful reading of the manuscript, i agree with the reviewers on the below issues raised*

1. *use of they and we is not appropriate for academic writing.*

We have edited the manuscript accordingly.

2. *professional editing is needed*

A professional editor helped us improve the manuscript.

3. *author need to look at the model specification and reduce the number of robust tests.*

We have corrected the model specification and dropped tests that were not critical.

**Authors’ Responses to Reviewer #1’s Comments**

*I suggest the use of models in research should be reflective of adequately, practically and competently measuring the constructs in the research. So even though the models used here are scientifically rigorous I suggest a* ***non-linear model*** *would have been more adequate, practical and competent.*

Thank you for the comment. Our default measure of crossblockholding is an indicator—that is, whether a firm in a year was cross-blockheld by any institutional investors in any quarter in a fiscal year and 0 otherwise. We cannot include a nonlinear term of an indicator variable in our base specification. Moreover, the academic editor and the reviewer #2 asked us to take off some robustness tests. So, in the interest of space, we could not consider a nonlinear mopdel.

**Authors’ Responses to Reviewer #2’s Comments**

1. *Abstract section need to be revised especially the use of the word ‘****They’****.*

We read <https://journals.plos.org/plosone/s/submission-guidelines> and do not see PLOS One’s preference on whether the abstract should be written in the first person or second person. Most journals prefer the abstract to be in the third person. The reader thus receives the impression that the journal—and not the authors—is talking to the reader. We thus have written the abstract in the “third person.” Should you prefer us to adopt “first person” and use “we” in place of “they,” we will comply.

2. *Is the benefits of CSR only less tangible to investors in short term? What about the long-run effects/benefits of CSR.*

Thank you for this thoughtful question. Investors become aware of a firm’s CSR activities/initiatives and their concomitant performance only when the firm discloses the CSR report each year. Therefore, we reason that the investors cannot appropriate the benefits of CSR in the short-term of less than a year. However, the benefits can carry over to the longer term and our manuscript measures a firm’s CSR performance in year *t* + 2 and average over two years (*t* + 1 and *t* + 2) and over three years (*t* + 1, *t* + 2, and *t* + 3).

3. *Suggest that this statement read as ‘****In this test, we first find that firms under greater institutional cross-blockholding receive less attention from investors****…………..’*

Thank you for suggesting this edit. We have tracked this edit in the uploaded manuscript.

4. *Authors should try as much as possible to* ***avoid the use of the word ‘we’****. Example can be found in this statement ‘We require observations to satisfy the following criteria:…….’*

Thank you for asking this edit. We have avoided using “we” wherever such avoidance did not hurt the flow and did not make the reader question “who did this.”

5. *‘Finally, we use overall CSR score (CSR), overall CSR strengths and concerns scores (STR and CON),……’I suggest that authors revise the statement especially to make it clear as to* ***what STR and CON represent respectively*** *in the statement.*

We have revised the text. Thank you.

6. *Authors should provide* ***the justification for the exclusion of observations if investors hold shares less than 5% of the firms’ total outstanding****.*

Revised; thank you.

7. *Authors need to* ***look at the model specification (i.e. eq. 1) again****.*

Corrected; thank you.

8. *Authors have done so many robustness test which distort the flow on what the study seek to achieve. I suggest authors* ***perform only robustness test on the key study objective****.*

Thank you for suggesting. We have excluded from the revised manuscript the tests for alternate explanations. That is, dropped the subsection 5.2 and the associated Table 12.

9. *Authors should provide* ***strong policy implications of the study.***

We absolutely agree and thank you for asking us to provide strong policy implications. We have added the text now.

10. *An editing help from someone with full professional proficiency in English is strongly recommended.*

Thank you again! We used the service of a sharp English editor to finesse the English. You will see tracked edits throughout the revised manuscript.
